# Surgical outcomes of very-early-onset ulcerative colitis: retrospective comparative study with older pediatric patients

**DOI:** 10.1007/s00383-024-05662-8

**Published:** 2024-03-07

**Authors:** Takashi Fumita, Keita Terui, Ryohei Shibata, Ayako Takenouchi, Shugo Komatsu, Satoru Oita, Hiroko Yoshizawa, Yuichi Hirano, Yusaku Yoshino, Takeshi Saito, Tomoro Hishiki

**Affiliations:** 1 Department of Pediatric Surgery, Graduate School of Medicine, Chiba University, 1-8-1 Inohana, Chuo-ku, Chiba, 260-8677 Japan; 2 Department of Pediatric Surgery, Chiba Children’s Hospital, Chiba, Japan

**Keywords:** Inflammatory bowel diseases, Pediatric ulcerative colitis, Early-onset, Ileostomy, Catheter-related infection

## Abstract

**Purpose:**

The study compares the surgical outcomes of very-early-onset ulcerative colitis (VEO-UC), which is a rare disease diagnosed in pediatric patients < 6 years, with those of older pediatric patients with ulcerative colitis (UC).

**Methods:**

A retrospective observational study of 57 pediatric patients with UC was conducted at a single center. The study compared surgical complications and postoperative growth between the two groups.

**Results:**

Out of the 57 patients, 6 had VEO-UC, and 5 of them underwent total colectomy. Compared with the surgical cases of older patients with UC (*n* = 6), the rate of postoperative complications in patients with VEO-UC (*n* = 5) was not significantly different, except for high-output ileostomy (80% vs. 0% at 3 weeks postoperatively, *p* = 0.02). The rate of postoperative central venous catheter (CVC) placement at > 90 days was higher in patients with VEO-UC (100% vs. 17%, *p* = 0.02). The median change in the *Z*-score of height before and 2 years after colectomy was not significantly different between VEO-UC and older patients (1.1 vs. 0.3, *p* = 0.13).

**Conclusion:**

With regard to complications and outcomes, total colectomy for VEO-UC patients and that for older pediatric UC patients is comparable. However, high-output ileostomy and the long duration of CVC placement may pose management challenges.

## Introduction

Inflammatory bowel disease (IBD) is a chronic and acute inflammation of the gastrointestinal tract, categorized into Crohn’s disease, ulcerative colitis (UC), or unspecified. Twenty-five percent of patients with IBD first present during childhood or adolescence [1, 2]. Pediatric-onset IBD has more severe characteristics than adult-onset IBD [3]. For instance, 82% of the childhood-onset-UC patients and 47% of the adult-onset-UC patients presented with extensive colitis, and the need for colectomy after 10 years was reported in 41% of the childhood-onset-UC patients and 20% of the adult-onset-UC patients [4].

Very-early-onset IBD (VEO-IBD) is a rare pediatric IBD diagnosed before 6 years of age [5]. According to a population-based study in Ontario, Canada, the incidence rates of VEO-IBD and older pediatric IBD cases are 3.4 and 59.9 per 100,000 individuals, respectively [6]. Although the incidence of VEO-IBD is very rare, it has been increasing at an alarming rate, with an average yearly change in incidence between 1994 and 2009 of + 7.4%, compared with + 2.2% in adolescent-onset UC [6]. As a result, VEO-IBD has become an increasingly important issue in pediatric gastroenterology.

For convenience, VEO-IBD has been classified as Crohn’s disease, UC, and unclassifiable [6]. Many studies have recently elucidated medical therapy for very-early-onset UC (VEO-UC) [2, 5, 7]. However, information on surgical interventions for VEO-UC is limited, and the optimal surgical strategy can only be drawn on data from older children and adults with UC since there have been few studies specifically focused on VEO-UC [5, 6, 8, 9].

Therefore, our research question was whether the surgical outcomes of VEO-IBD were similar to those of older children with UC. In this study, we aimed to clarify the surgical outcomes, especially postoperative complications, management, and mid-term growth of VEO-IBD, by comparing them with those of older pediatric UC patients. This information will be useful to consider surgical indications for VEO-UC.

## Method

### Study design and patient selection

This retrospective comparative study included patients diagnosed with UC before the age of 16 years and managed at Chiba University Hospital from March 2007 to December 2022. The recruited patients with UC were divided into two groups: one comprising patients with VEO-UC diagnosed before the age of 6, and the other comprising older pediatric patients with UC onset at a later age. The surgical outcomes were compared between the two groups.

### Preoperative management of UC patients

Patients with UC were diagnosed based on the macroscopic and microscopic findings of upper esophagogastroduodenoscopy and total colonoscopy with terminal ileal intubation. These diagnoses were confirmed by pathological findings of surgical specimens in surgical cases. For VEO-UC cases, genetic testing was performed when feasible. The medical management of UC patients included steroids, immune suppressors, biological agents, and granulocytapheresis (GCAP), which were performed in accordance with the treatment guidelines established by The Japanese Society for Pediatric Inflammatory Bowel Disease. Biological drugs have been used at our institution since 2013.

### Surgical indication, procedure, and postoperative management

Surgical treatment was selected in cases of steroid dependence or resistance, poor response to biological agents or immunosuppressive drugs, or significant progression of anemia during treatment. Laparoscopic surgery with five ports was chosen; however, in cases where the patient was too small to provide sufficient working space, open surgery was chosen. Our standard surgical protocol involved a two-stage approach, with the first stage comprising a total colorectal resection, ileal pouch-anal anastomosis, and covered ileostomy which is created 30 cm proximal from anal anastomosis of the ileal pouch. The closure of the ileostomy was the second stage of the process. In patients with a worse general condition, ileostomy and/or a subtotal resection of the colon was performed during the first stage. After the colectomy, when sufficient output of ileostomy is confirmed, water intake and enteral nutrition were started. Antidiarrheal drugs were used at the surgeon’s discretion depending on the stool amount.

### Data collection

The patient clinical data extracted from the charts include sex; district of residence; area of lesion; time of diagnosis and discharge; pediatric ulcerative colitis activity index (PUCAI); use of 5-aminosalicylic acid; administration of steroid, immune suppressor, and biological drugs; use of GCAP; time of surgical intervention; surgical procedure; postoperative complications such as wound infection, pouchitis, adhesive small-bowel obstruction, anastomotic stricture, ileostomy prolapse, and outlet obstruction of ileostomy; amount of ileostomy output at 1, 2, 3, and 4 postoperative weeks; need for central venous catheter (CVC); incidence of catheter-related bloodstream infection (CRBSI); and *Z*-score of body height and weight. PUCAI is a scoring system of severity based on six items: abdominal pain, rectal bleeding, stool consistency of most stools, number of stools per 24 h, nocturnal stools, and activity level. PUCAI of less than 10 points, 15–30 points, 35–60, 35–60, and 65 or more represents remission, mild disease, moderate disease, and severe disease, respectively [10]. High output ileostomy was defined as the amount of ileostomy output of > 50 mL/kg/day [11].

### Statistical analysis

The summary statistics for categorical data are expressed as frequencies and proportions, whereas continuous variables are summarized using the mean and standard deviation or median and range. The Wilcoxon signed-rank and Wilcoxon rank-sum tests were used for comparing continuous variables, and the chi-square test or Fisher’s exact test was used for categorical variables. The Kaplan–Meier method and log-rank test were used to estimate and compare CVC placement days, the time from colectomy to discharge, the time from colectomy to ileostomy closure, and CRBSI-free times. Statistical significance was defined as a two-sided *p* < 0.05. All statistical analyses were performed using GraphPad Prism software (version 9; GraphPad Software Inc., San Diego, CA, USA).

### Ethical approval

The Research Ethics Committee of the Graduate School of Medicine at Chiba University (No. M10108) approved this study. As the study was retrospective and involved the use of de-identified data, the requirement for signed informed consent was waived. The details of the study were published on an institutional website, and individuals had the right to decline their participation. The study was conducted in accordance with the principles of the Declaration of Helsinki and the ethical guidelines for medical and health research involving human subjects.

## Results

### Baseline characteristics

A total of 57 pediatric UC patients were treated at our institution, of which 6 patients had VEO-UC (10%). The demographic data of the VEO-UC and older pediatric UC patients are presented in Table 1. The age at diagnosis was significantly lower in the VEO-UC group (1.1 vs. 12.4 years, *p* < 0.0001). The colectomy rates among the VEO-UC and older pediatric UC patients were 83% (5 out of 6 cases) and 12% (6 out of 51 cases), respectively (*p* = 0.0006). The proportion of patients from other prefectures was significantly higher in the VEO-UC group (50% vs. 0%, *p* = 0.0007).

**Table 1:** Characteristics of patients with pediatric ulcerative colitis

	VEO-UC (*n* = 6)	Older pediatric UC (*n* = 51)	*p* value
Age at diagnosis	1.1 (0.6–4.0)	12.4 (6.2–15.7)	** < 0.0001**
Sex (male: female)	1:5	28:23	0.10
Patients introduced from other prefectures	3 (50%)	0 (0%)	**0.0007**
Type (rectum/left side/total colon)	0/0/6	1/16/34	0.24
PSL, *n* (%)	6 (100%)	43 (84%)	0.58
AZA or 6-MP, *n* (%)	3 (50%)	31 (61%)	0.68
TAC or CyA, *n* (%)	3 (50%)	13 (25%)	0.33
GCAP, *n* (%)	0 (0%)	15 (29%)	0.32
BIO, *n* (%)	3 (50%)	9 (17%)	0.10
Colectomy, *n* (%)	5 (83%)	6 (12%)	**0.0006**

*VEO-UC* very-early-onset ulcerative colitis, *PSL* prednisolone, *AZA* azathioprine, *6-MP* 6-mercaptopurine, *TAC* tacrolimus, *CyA* cyclosporin A, *GCAP* granulocyte apheresis, *BIO* biologic agent

*p* value corresponds to the comparison of VEO-UC and older pediatric UC, and *p* value (< 0.05) is shown in bold

### Comparison of surgical outcomes between VEO-IBD and older pediatric UC

Table 2 shows the characteristics of the VEO-UC and older pediatric UC patients who underwent total colectomy. Ages at diagnosis (0.9 vs. 12.2 years, *p* < 0.0001) and colectomy (2.8 vs. 14.4 years, *p* < 0.0001) were significantly lower in the VEO-UC group. There were no statistically significant differences in sex, total steroid use until colectomy, area of lesion, and time from UC diagnosis to colectomy.

**Table 2:** Characteristics of patients who underwent colectomy

	VEO-UC (*n* = 5)	Older pediatric UC (*n* = 6)	*p* value
Age at diagnosis (years)	0.9 (0.6–2.0)	12.2 (10.2–15.1)	** < 0.0001**
Gender (male:female)	1:4	4:2	0.24
Total steroid use until colectomy (mg/kg)	255.9	145.2	0.23
Area of lesion (rectum/left side/total colon)	0/0/5	0/1/5	1.0
Age at colectomy (years)	2.8 (1.1–3.5)	14.4 (12.2–16.7)	** < 0.0001**
PUCAI at colectomy	40 (35–65)	35 (10–65)	0.33
Time from UC diagnosis to colectomy (years)	2.0 (0.2–2.6)	1.5 (0.9–6.5)	0.52

*VEO-UC* very-early-onset ulcerative colitis, *PUCAI* pediatric ulcerative colitis activity index

*p* value corresponds to the comparison of VEO-UC and older pediatric UC, and *p* value (< 0.05) is shown in bold

**Fig. 1:**
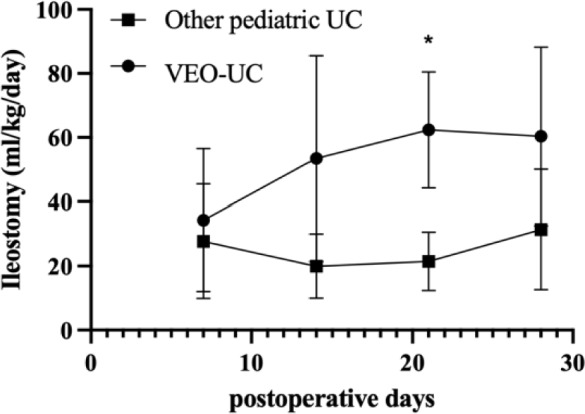
Postoperative output of ileostomy. The amount of ileostomy output between the two groups was assessed using the Wilcoxon signed-rank test. Plots represent the median of the amount of ileostomy output and bars represent standard errors. *p = 0.0009

**Fig. 2:**
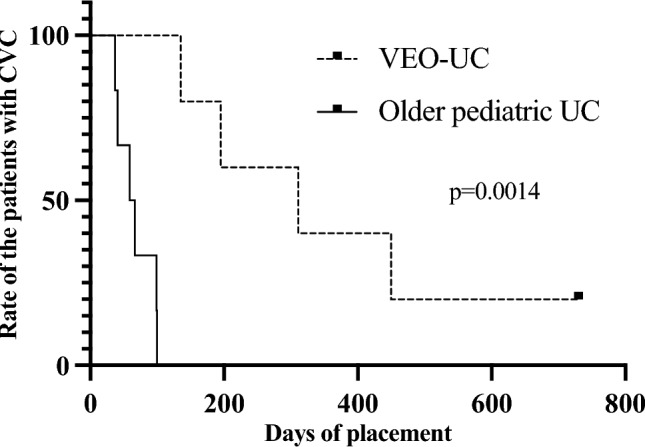
Kaplan–Meier curves of CVC removal within 2 years from colectomy. *CVC* central venous catheter

**Fig. 3:**
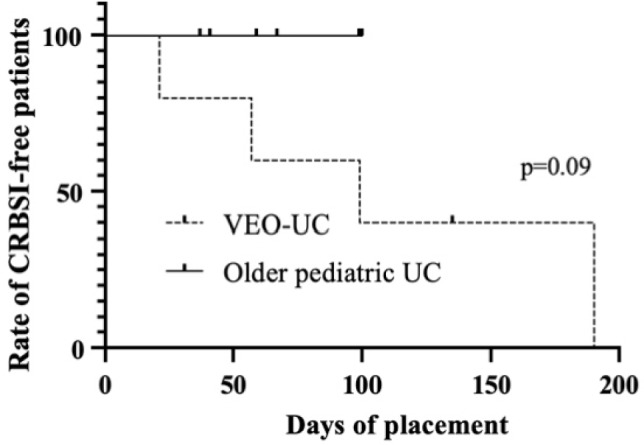
Kaplan–Meier curves of catheter-related bloodstream infection. *CRBSI* catheter-related bloodstream infection

We compared the surgical outcomes between the VEO-IBD and older pediatric UC patients (Table 3). There were no statistically significant differences in postoperative complications within 2 years of colectomy, except for high-output ileostomy. Time from colectomy to discharge (*p* = 0.001) and to ileostomy closure (*p* = 0.02) was significantly longer in VEO-UC patients. The rate of high-output ileostomy was significantly higher in patients with VEO-IBD 3 weeks postoperatively (80% vs. 0%, *p* = 0.02). Figure 1 illustrates the postoperative changes in the ileostomy output of the VEO-UC and older pediatric UC patients. Patients with VEO-IBD had a significantly higher ileostomy output at 3 weeks postoperatively (62 vs. 21 mL/kg, *p* < 0.0009). The median rate of postoperative CVC placement of > 90 days was significantly higher in the VEO-UC group (100% vs. 17%, *p* = 0.02). The Kaplan–Meier curve of CVC placement showed significantly longer duration in VEO-UC patients (*p* = 0.0014) (Fig. 2). The number of CRBSIs/1000 catheter days was not significantly different between the two groups (2.0 vs. 0, *p* = 0.08). The Kaplan–Meier curve of the CRBSI showed no between-group differences (*p* = 0.09) (Fig. 3).

**Table 3:** Surgical complications and outcomes of patients with VEO-UC and older pediatric patients with UC

	VEO-UC (*n* = 5)	Older pediatric UC (*n* = 6)	*p* value
Wound infection, *n* (%)	0 (0%)	0 (0%)	1.0
Pouchitis within 2 years after colectomy, *n* (%)	1 (33%)*	2 (33%)	1.0
Adhesive small-bowel obstruction within 2 years after colectomy, *n* (%)	0 (0%)	1 (17%)	1.0
Anastomotic stricture within 2 years after colectomy, *n* (%)	0 (0%)	1 (17%)	1.0
Prolapse of ileostomy within 2 years after colectomy, *n* (%)	1 (20%)	0 (0%)	1.0
Pelvic abscess within 2 years after colectomy, *n* (%)	0 (0%)	0 (0%)	1.0
Ileostomy outlet obstruction within 2 years after colectomy, *n* (%)	0 (0%)	2 (33%)	0.45
Ileostomy output > 50 mL/kg at 1 week postoperatively, *n* (%)	1 (20%)	1 (17%)	1.0
Ileostomy output > 50 mL/kg at 2 weeks postoperatively, *n* (%)	2 (40%)	0 (0%)	0.18
Ileostomy output > 50 mL/kg at 3 weeks postoperatively, *n* (%)	4 (80%)	0 (0%)	**0.02**
Ileostomy output > 50 mL/kg at 4 weeks postoperatively, *n* (%)	4 (80%)	1 (17%)	0.08
Postoperative duration of CVC placement > 90 days	5 (100%)	1 (17%)	**0.02**
CRBSI within 30 days postoperatively, *n* (%)	1 (20%)	0 (0%)	1.0
Number of CRBSIs/1000 catheter days	2.0 (0–9.6)	0 (0–0)	0.08
Time from colectomy to discharge (days)	198 (83–561)	46 (42–69)	**0.001**
Time from colectomy to ileostomy closure (years)	1.4 (0.2–6.2)	0.3 (0.1–0.4)	**0.02**
Duration of postoperative follow-up (years)	6.4 (2.6–14.6)	8.5 (4.8–11.2)	0.73

*VEO-UC* very-early-onset ulcerative colitis, *CVC* central venous catheter, *CRBSI* catheter-related bloodstream infection

*p* value corresponds to the comparison of VEO-UC and older pediatric UC, and *p* value (< 0.05) showed with bold

*2 cases were removed from the analysis because one was without an ileal pouch and the other was not closed ileostomy within 2 years

**Fig. 4:**
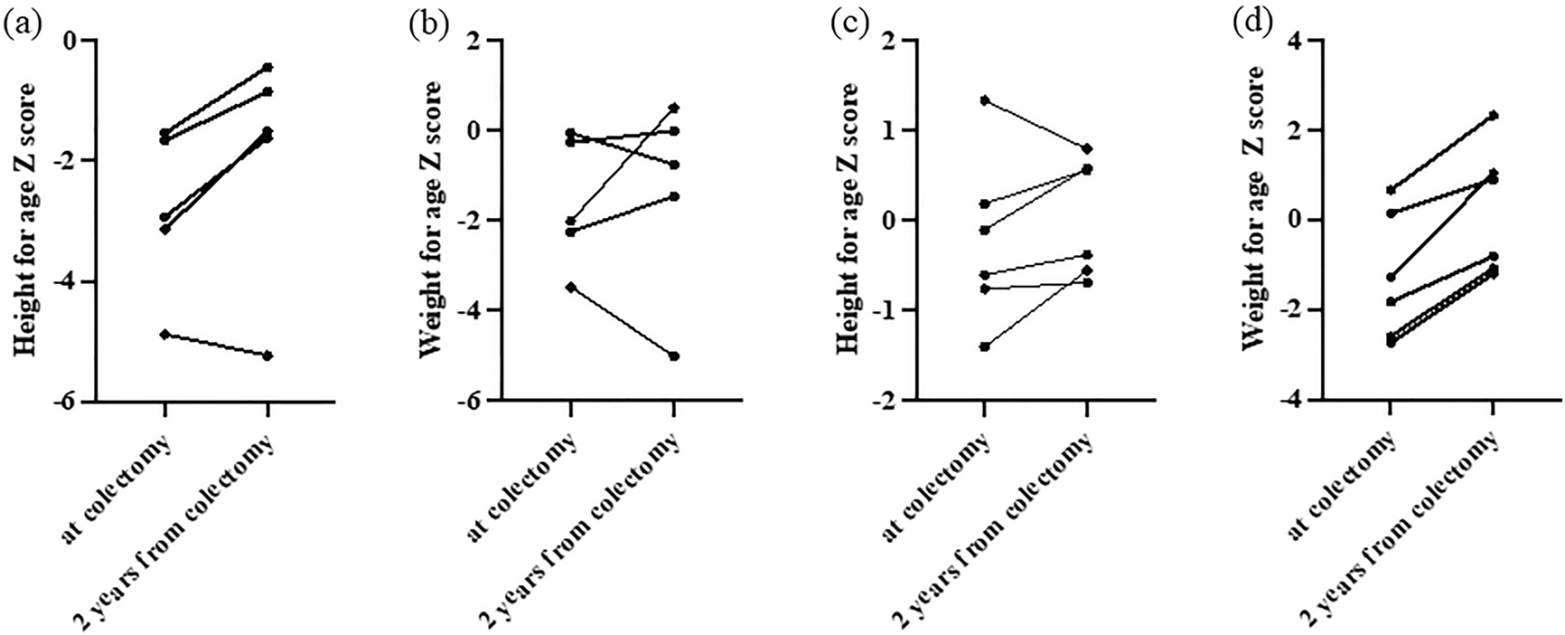
Changes in height and weight for age *Z*-scores at colectomy and 2 years from colectomy. **a**, **b** Height and weight for age *Z*-scores of patients with very-early-onset ulcerative colitis. **c**, **d** Height and weight for age *Z*-scores of older patients with ulcerative colitis

Table 4 presents a comparison of *Z*-scores for body height and weight, which were measured at colectomy and 2 years postoperatively, between the VEO-UC and older pediatric UC patients. The results indicate that the height *Z*-scores of the VEO-UC patients were significantly lower at colectomy (− 2.9 vs. − 0.4, *p* = 0.005) and 2 years postoperatively (− 1.5 vs. 0.1, *p* = 0.04). However, there were no significant between-group differences in the change in *Z*-scores for height (1.1 vs. 0.3, *p* = 0.13) and weight (0.8 vs. 1.5, *p* = 0.11) before and 2 years after colectomy. Figure 4 shows these changes for each patient with VEO-UC.

**Table 4:** Height and weight *Z*-scores at colectomy and 2 years after colectomy

	VEO-UC (*n* = 5)	Other pediatric UC (*n* = 6)	*p* value
Height *Z*-score at colectomy	− 2.9 ( − 4.9 to − 1.5)	− 0.4 ( − 1.4 to 1.3)	**0.005**
Height *Z*-score after 2 years from colectomy	− 1.5 ( − 5.2 to − 0.4)	0.1 ( − 0.7 to 0.8)	**0.04**
Change in height *Z*-score before and 2 years after colectomy	1.1 ( − 0.3 to 1.6)	0.3 ( − 0.5 to 0.8)	0.13
Weight *Z*-score at colectomy	− 2.0 ( − 3.5 to − 0.1)	− 1.5 ( − 2.7 to 0.7)	0.69
Weight *Z*-score after 2 years from colectomy	− 0.8 ( − 5.0 to 0.5)	0.0 ( − 1.2 to 2.3)	0.19
Change in weight *Z*-score before and 2 years after colectomy	0.8 ( − 1.5 to 2.5)	1.5 (0.8 to 2.3)	0.11

*VEO-UC* very-early-onset ulcerative colitis, *UC* ulcerative colitis

*p* value corresponds to the comparison of VEO-UC and older pediatric UC, and *p* value (< 0.05) is shown in bold

### Details of VEO-UC cases

Table 5 shows the details of VEO-UC patients. Of the six patients, five had an infantile onset, defined as onset before 2 years of age [12], and all underwent colectomy. All VEO-UC patients had total colitis. Two patients (Cases 4 and 6) underwent gene sequencing for primary immune deficiency including 20 genes [13], which revealed a nonsense mutation of STAT1 at chr2:190,987,050 (c. 1116T > C) in Case 6. Two patients (Cases 4 and 5) underwent flow cytometry to rule out primary immune deficiency, which revealed no abnormalities. Surgical cases of VEO-UC underwent full preoperative medical treatment, including steroids, immune suppressors, and biologic agents, except for two patients owing to parental wishes. The median time from UC diagnosis to colectomy was 2.0 years (range 0.2–2.4 years). The non-surgical VEO-UC case (Case 2) had been undergoing medical therapy for 11 years since the age of 4. Three patients underwent our standard two-stage surgery. Case 1 underwent subtotal resection of the colon first because of poor preoperative condition. Case 6 underwent ileostomy first because preservation of the colon was attempted before confirming the diagnosis. The median time from colectomy to ileostomy closure was 1.4 years (range 0.2–6.2 years).

**Table 5:** Details of patients with very-early-onset ulcerative colitis (VEO-UC

Case	Sex	Age at diagnosis (years)	Area of lesion	PUCAI at first visit	Preoperative treatment					Duration of drug treatment (years)	PUCAI at colectomy	Age at colectomy (years)	Stage procedure	Time from colectomy to ileostomy closure (years)	Age at CVC removal (years)
					5-ASA	PSL	AZA	TAC	BIO						
1	F	0.9	Total colon	45	+	+				0.2	60	1.1	2	0.6	1.4
2	F	4.0	Total colon	40	+	+				11	–	–	–	–	–
3	M	1.3	Total colon	40	+	+	+	+	+	2.2	40	3.5	2	0.2	4.3
4	F	0.6	Total colon	20	+	+	+	+	+	2.4	35	3.0	2	6.2	Not yet (9.8)
5	F	2.0	Total colon	35	+	+	+	+	+	1.2	65	3.2	2	2.3	4.7
6	F	0.9	Total colon	30	+	+				2.0	35	2.9	3	1.4	3.2

*PUCAI* pediatric ulcerative colitis activity index, *5-ASA* 5-acetylsalicylic acid, *PSL* prednisolone, *AZA* azathioprine, *TAC* tacrolimus, *BIO* biologic agent, *CVC* central venous catheter

## Discussion

This study aimed to compare the surgical outcomes in patients with VEO-UC with those of older pediatric UC patients. Our study found that (1) VEO-UC patients did not have a significant difference in surgical complication rates compared to older pediatric UC patients, except for high-output ileostomy; (2) VEO-UC patients had a higher ileostomy output, resulting in a prolonged duration of ileostomy and CVC placement; and (3) postoperative growth recovery of patients with VEO-UC might be comparable to that of pediatric patients with UC.

The colectomy rate for pediatric UC is reportedly higher than that for adult UC. Siow et al. reported that the 10-year colectomy rates in pediatric patients range from 30 to 40% compared with 15–25% in adults [14]. The present study showed that the colectomy rate of the VEO-UC group was significantly higher than that of the older pediatric patients with UC. However, half of our patients with VEO-UC were referred from other distant prefectures for undergoing surgery. This patient selection bias might have influenced the higher colectomy rate of the VEO-UC group in our cohort. Benchimol et al. reported that the colectomy rate in VEO-UC patients was lower than that in pediatric UC patients over 10 years of observation (8.2% vs. 17.7%) [6]. A study focusing on IBD by Cucinotta et al. reported that the surgical risk of patients with VEO-IBD was significantly higher than that of older pediatric patients with IBD (32% vs. 14%) after 10 years of observation [15]. Comparison between the colectomy rate of the VEO-UC and older pediatric UC remains controversial.

A previous study has shown that younger patients with VEO-IBD have a shorter duration from diagnosis to colectomy: Among patients who develop VEO-IBD during the first year of life, approximately one-third require surgical treatment within the first year after onset [16]. In the present study, the duration from diagnosis to colectomy was not different from that in older pediatric UC patients, although the VEO-UC patients who underwent surgery at our institution were diagnosed with UC at < 2 years of age. Duration from diagnosis to colectomy may have been affected by progress in medical therapies and disease severity of the included patients.

The surgical complications of UC include pelvic sepsis, anastomotic leakage, anastomotic stricture, and pouchitis, while complications of ileostomies include outlet obstruction, prolapse, and high-output ileostomies. General surgical complications include wound infection and small-bowel obstruction [17]. Pediatric patients with UC appear to have a different clinical course than adults. Tan Tanny et al. [18] found a higher incidence of pouchitis and lower rates of pelvic infection in this population. Similarly, Rinawi et al. [19] suggested that pouchitis is more common in younger patients with pediatric UC. In terms of surgical complications in the pediatric population, Knod et al. [20] reported rates of 17–50% for bowel obstruction, 5–13% for anastomotic leak, 9–27% for stricture, and 24–29% for pouchitis. The present study revealed that the rate of surgical complications in patients with VEO-UC did not differ from that in older pediatric UC patients, except for high-output ileostomy. The complication rate of VEO-UC in our study was comparable to that of pediatric UC in previous studies [18, 20].

High-output ileostomy occurs when fluid loss through ileostomy exceeds the patient’s compensatory mechanisms, resulting in dehydration, acute kidney failure, and even death. The causes of this condition are not yet fully understood [21]. Knod et al. [20] reported that the rates of dehydration after colectomy among pediatric UC patients aged over and under 11 years were 5.3% and 15.4%, respectively. These data indicate that dehydration is more common in younger patients, which supports our findings. It was very interesting that there was a difference in the amount of ileostomy by age, however, details could not be assessed because the management for high-output ileostomy was not protocolized.

The treatment of high-output ileostomy involves replenishing lost electrolytes and fluids, along with measures to decrease defecation. Therefore, prolonged high-output ileostomy results in a longer duration of placement of a CVC. Actually, a significantly longer duration of CVC replacement was observed in VEO-UC in the present study. The long duration of CVC replacement often results in the more frequent occurrence of CRBSI. In the present study, although there was no significant difference in a number of CRBSIs/1000 catheter days, patients with VEO-UC had 2 CRBSIs/1000 catheter days while the older pediatric UC patients had no CRBSI. Shibata et al. reported that the incidence of CRBSI in IBD and non-IBD adults was 13.2 and 0.40 infections per 1000 catheter days, respectively [22]. Therefore, the long duration of CVC replacement against UC patients is apparently a risk of CRBSI. Early detection and appropriate treatment of CRBSI are essential.

In our study, we found that patients with VEO-UC had a significantly lower *Z*-score for height at the time of colectomy compared with older patients with UC. This suggests that the growth of VEO-UC patients may be more adversely affected by the disease compared with the growth of older patients, as there was no significant difference in the time from UC diagnosis to colectomy between the two groups. Furthermore, although the *Z*-score of height 2 years after colectomy was also significantly lower in patients with VEO-UC, the change in the height *Z*-scores before and after colectomy was comparable to that in older pediatric patients with UC. The effect on postoperative growth after surgery for patients with VEO-UC and for older pediatric patients with UC was not significantly different. Nicholls et al. reported that the growth velocity of height is accelerated nearly two-fold after surgery in pediatric patients [23]. Moreover, Sako et al. [24] reported that a growth “catch-up” has been obtained in 14 out of 15 pediatric UC patients. There are no detailed data on pre- and postoperative *Z*-scores of anthropometric measurements in pediatric UC patients; therefore, to our knowledge, this is the first study to show the effect of surgery on postoperative growth using *Z*-scores in pediatric UC patients.

This study has some limitations. First, VEO-IBD is a complex and heterogeneous disease, and the diagnosis of VEO-UC may change as the patient ages [25]. Rialon et al. [26] reported that 24% of patients originally diagnosed with UC or IBD-unclassified were later reclassified as having Crohn’s disease. In this study, the diagnosis of VEO-UC was based on endoscopic findings, and genetic testing was not performed in all cases, meaning that underlying immunodeficiency and monogenic etiologies were not thoroughly ruled out. However, small intestinal lesions were not detected in any of the VEO-UC cases, and the diagnosis of VEO-UC did not change during a median postoperative follow-up period of 6.4 years. Lastly, we did not compare the postoperative quality of life, which is one of the most important surgical outcomes. Future studies are needed to address this aspect.

## Conclusion

The indication of total colectomy for refractory VEO-UC patients may be considered equally as that for older pediatric UC patients in terms of the rate of complications and outcomes. However, management of high-output ileostomy and a long duration of CVC placement duration may pose challenges.

## Data Availability

No datasets were generated or analysed during the current study.
